# Layer-by-layer heparinization of decellularized liver matrices to reduce thrombogenicity of tissue engineered grafts

**DOI:** 10.18053/jctres.201501.004

**Published:** 2015-07-19

**Authors:** Bote G Bruinsma, Yeonhee Kim, Tim A Berendsen, Sinan Ozer, Martin L Yarmush, Basak E Uygun

**Affiliations:** 1 Center for Engineering in Medicine, Department of Surgery, Massachusetts General Hospital, Harvard Medical School, and the Shriners Hospitals for Children, Boston, Massachusetts, United States.; 2 Department of Surgery (Surgical Laboratory), Academic Medical Center, University of Amsterdam, Amsterdam, the Netherlands; 3 Department of Biomedical Engineering, Rutgers University, Piscataway, New Jersey, United States.

**Keywords:** Tissue engineering, decellularization, recellularization, heparinization, thrombogenicity, hemocompatibility, transplantation

## Abstract

**Background:**

Tissue–engineered liver grafts may offer a viable alternative to orthotopic liver transplantation and help overcome the donor organ shortage. Decellularized liver matrices (DLM) have a preserved vasculature and sustain hepatocellular function in culture, but graft survival after transplantation remains limited due to thrombogenicity of the matrix.

**Aim:**

To evaluate the effect of heparin immobilization on DLM thrombogenicity.

**Methods:**

Heparin was immobilized on DLMs by means of layer-by-layer deposition. Grafts with 4 or 8 bilayers and 2 or 4 g/L of heparin were recellularized with primary rat hepatocytes and maintained in culture for 5 days. Hemocompatibility of the graft was assessed by ex vivo diluted whole-blood perfusion and heterotopic transplantation.

**Results:**

Heparin was deposited throughout the matrix and the heparin content in the graft was higher with increasing number of bilayers and concentration of heparin. Recellularization and in vitro albumin and urea production were unaffected by heparinization. Resistance to blood flow during ex vivo perfusion was lower with increased heparinization and, macroscopically, no clots were visible in grafts with 8 bilayers. Following transplantation, flow through the graft was limited in all groups. Histological evidence of thrombosis was lower in heparinized DLMs, but transplantation of DLM grafts was not improved.

**Conclusions:**

Layer-by-layer deposition of heparin on a DLM is an effective method of immobilizing heparin throughout the graft and does not impede recellularization or hepatocellular function in vitro. Thrombogenicity during ex vivo blood perfusion was reduced in heparinized grafts and optimal with 8 bilayers, but transplantation remained unsuccessful with this method.

**Relevance for patients:**

Tissue engineered liver grafts may offer a viable solution to dramatic shortages in donor organs

## 1. Introduction

Liver transplantation remains the only effective treatment of end-stage liver disease. Although highly successful, this procedure is severely limited by the shortage of donor organs with thousands dying annually in the United States that could potentially have benefited from a liver transplant [1]. Hence, alternative methods to supply viable cells and tissues to support hepatic function in end-stage patients are urgently needed.

Tissue engineering approaches provide the tools to create constructs that serve as substitutes for donor livers and potentially address the significant gap in the number of donated livers [2]. However, advances in liver tissue engineering have been hindered by the lack of a suitable scaffold architecture that enables delivery of the nutrients and removal of waste materials from the cells that are seeded inside the construct. Whole-organ engineering is a recently-emerged approach that uses the native organ structure as the scaffold. In whole-liver engineering donor livers are decellularized by perfusion of detergents into the vasculature to create a whole-organ scaffold, which retains the native extracellular matrix (ECM) composition. Preservation of important ECM components and the vascular architecture facilitates cell engraftment and tissue development [3, 4], perfusion of the graft and surgical implantation. We recently developed a protocol to create whole-liver grafts by repopulating a decellularized liver matrix (DLM) with primary rat hepatocytes [5]. These grafts retain liver-specific function for up to 10 days during in vitro perfusion culture. Long-term survival following transplantation, however, remains hindered by thrombogenicity of the grafts, where exposed collagen promotes coagulation upon blood reperfusion. Preventing thrombus formation in the DLM graft is a key challenge to overcome before long-term post-transplantation viability can be attempted [6].

Endothelial cell seeding can reduce coagulation, but coverage of the vasculature is often incomplete, leaving defects in the vascular lining that remain thrombogenic. Systemic anticoagulation is also suboptimal, putting the recipient at risk of hemorrhagic complications and is not ideal for long–term maintenance. In tissue engineering, one strategy to improve hemocompatibility of devices that come in direct contact with blood and/or plasma is through surface modification with bioactive agents, such as heparin [7]. Heparin acts by binding to the protein antithrombin III, which then binds to its target molecules thrombin and coagulation factor Xa with high affinity [8]. It has been used in tissue engineering to modify biomaterials for thromboresistance either by direct blending into the biomaterial [9] or through coating of the graft surface [8]. Surface coating with heparin has been achieved by covalent immobilization [10, 11] and by surface adsorption [12-14]. In order to achieve loading of a high quantity of heparin and its release to the environment, heparin coating using layer-by-layer self assembled films has been reported [13, 15]. Layer– by–layer (LbL) self–assembly technique involves sequential deposition of a positively charged polyelectrolyte and heparin, which is negatively charged, on the biomaterial surface creating a multilayer film held together by electrostatic force. Such an approach was reported to delay blood coagulation when used to modify liver decellularized liver matrix scaffolds, and improve survival following transplantation in both a rodent and porcine model [15, 16].

**Figure 1.**
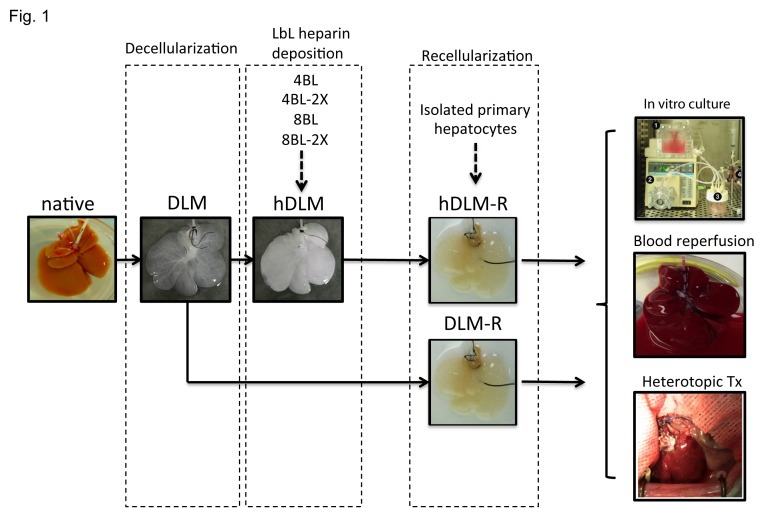
Schematic overview of the experiments. Native livers were decellularized (DLM) and coated with an increasing number of heparin bilayers (BL) to form heparinized DLM (hDLM), heparinized and unmodified (h)DLM were seeded with primary hepatocytes to form recellularized DLM ((h)DLM–R) and maintained in vitro perfusion culture, ex vivo diluted whole blood reperfusion or transplanted heterotopically.

In an attempt to improve hemocompatibility of the recellularized liver grafts and extend the recipient survival time when transplanted, we employed a layer-by-layer self-assembly technique creating a multilayer heparin coating of the ECM of our previously reported DLM. We examine the effects of heparin coating on hepatic function during in vitro perfusion culture of recellularized liver grafts. Moreover, ex vivo diluted whole-blood perfusion was used to examine thrombogenicity and grafts were heterotopically transplanted to examine in vivo post– reperfusion viability (Figure 1).

## 2. Materials and methods

### 2.1. Animals and liver procurement

All animal care, handling and surgical procedures were performed in accordance with the guidelines set by Institutional Animal Care and Use Committee (IACUC) at Massachusetts General Hospital. Female Lewis rats (Charles River Laboratories, Wilmington, MA, USA) weighing between 175–200 g were used for primary hepatocyte isolation, liver donation and liver transplant recipients. In brief for liver procurement, rats were anesthetized with 0.3 L/min of a 3% (v/v) isofluorane/ 97% oxygen gas mixture (Forane, Baxter, Deerfield, IL, USA). The abdomen was exposed by transverse incision and the surrounding hepatic ligaments were dissected. The portal vein (PV) was mobilized, and the gastroduodenal and splenic veins were ligated using 7-0 prolene suture (Ethicon, Somerville, NJ, USA) and dissected. The hepatic artery was ligated and transected. The infrahepatic inferior vena cava (IHIVC) was mobilized and elongated by ligation and dissection of the adrenal vein, lumbar plexus, right renal artery and right renal vein. The liver was excised from its recess and the suprahepatic inferior vena cava (SHIVC) was identified and cut. A cuff technique was applied to the PV and IHIVC according to the method described by Kamada et al. [17]. The procured liver was gently flushed with 0.9% NaCl solution and stored in 0.9% NaCl at –80 °C until utilized for decellularization. Three livers were used for every experiment and every group.

### 2.2. Preparation of DLM scaffolds

The liver was thawed at room temperature and washed by portal vein perfusion with phosphate buffered saline (PBS) overnight at a flow rate of 1.0 mL/min to clear residual blood from the organ. Then, the liver was perfused with increasing concentrations of sodium dodecyl sulfate (SDS) (Sigma-Aldrich, St. Louis, MO, USA) dissolved in distilled H_2_O for 60 h, starting with 0.01% (w/v) SDS for 24 h, with 0.1% (w/v) SDS for 24 h, and 0.2% (w/v) SDS for 12 h. Next, the liver was perfused with 0.1% (v/v) Triton X-100 (Sigma-Aldrich) in distilled H_2_O for 30 min to remove the residual components and rinsed with PBS for at least 2 h. The decellularized liver matrix was sterilized with PBS containing 0.1% (v/v) peracetic acid and 4% (v/v) ethanol (Sigma-Aldrich) for 3 h and rinsed with sterile PBS extensively. The sterilized DLM was stored in PBS supplemented with 250 µg/mL amphotericin B (Sigma-Aldrich), 200 U/mL penicillin and 200 mg/mL streptomycin (Invitrogen, Carlsbad, CA, USA) for up to 7 days.

### 2.3. Layer–by–layer heparin immobilization on DLM scaffolds

**Figure 2.**
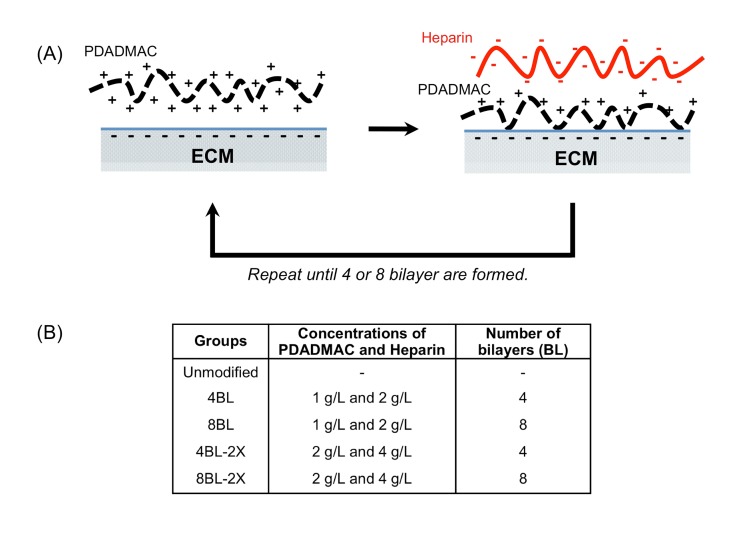
Heparin deposition on DLM scaffolds. (A) Schematic representation of layer-by-layer assembly of positively (PDADMAC) and negatively (heparin) charged polyelectrolytes on DLM scaffolds. (B) Experimental groups.

Sodium heparin (heparin) and low molecular weight poly (diallyldimethyl ammonium chloride) (PDADMAC) (Sigma-Aldrich) were used to coat heparin on to the matrix surfaces in a layer–by–layer (LbL) immobilization approach (Figure 2A), creating heparinized DLM (hDLM) scaffolds as described by Bao et al. [15]. Sodium heparin and PDADMAC were dissolved in PBS (pH 7.4) and used as the anionic and cationic polyelectrolytes (PEs). The sterilized DLM scaffold was flushed with PBS for 10 min prior to LbL assembly. To form one bilayer of PDADMAC/heparin, the DLM scaffold was first perfused with PDADMAC solution for 10 min, followed by 10 min of static incubation to allow binding of PDADMAC to the DLM surface. Unbound PDADMAC was removed via perfusion with PBS for 10 min. The DLM scaffold was then perfused with heparin solution for 10 min and heparin was allowed to bind to the PDADMAC layer by static incubation for 10 min. Unbound heparin was removed by perfusion with PBS for 10 min. These steps were repeated until the desired number of bilayers was formed. All perfusions were performed at a flow rate of 4 mL/min through the portal vein. The concentrations of PEs and the numbers of bilayers used to prepare hDLM scaffolds are given in Figure 2B.

### 2.4. Determination of heparin content of the scaffolds

The amount of heparin deposition in hDLM scaffolds was quantified by a hexosamine assay, which colorimetrically determines glucosamine concentration [18]. The entire DLM or hDLM scaffolds were lyophilized and cut into small pieces prior to the assay. The scaffold and heparin standards (0-4 mg) were hydrolyzed with 1 mL of 6 M hydrochloric acid (HCl) solution at 105 °C for 6 h in sealed glass test tubes. Hydrolyzed samples were neutralized and dried under vacuum in the presence of sodium hydroxide pellets for 2 h. The samples were dissolved in 1.25 mL of 18 MΩ-cm deionized water and 1 mL of sample was mixed with 1 mL of 4% acetyl acetone in 1.25 M sodium carbonate. The reaction mixture was incubated at 95 °C for 1 h to convert glucosamine into pyrrole derivatives. Then, 1.0 mL Ehrlich’s reagent (2.66% *p*-dimethylamino benzaldehyde in 3 M HCl and 47.5% ethanol) and 5 mL of 95% ethanol was added into the reaction mixture to develop a stable reddish purple color by condensation of pyrroles. The absorbance of the resulting solution was measured spectrophotometrically at 527 nm.

### 2.5. Primary rat hepatocyte isolation

Primary rat hepatocytes were isolated using the two–step perfusion protocol as described previously [19].

### 2.6. Hepatocyte seeding of the scaffolds

Hepatocytes were seeded by perfusion to form a recellularized (h)DLM ((h)DLM–R). The median lobe of heparinized or unmodified DLM scaffold was resected and connected to the perfusion system through portal vein cannulation. The IHIVC and SHIVC were left open for passive outflow. The liver was kept immersed in the perfusate in a perfusion chamber while perfused through the portal vein. A total volume of 100 mL of medium was recirculated in the perfusion system. Portal pressure was maintained between 10-15 cmH_2_O. The perfusate consisted of high-glucose Dulbecco’s modified Eagle’s medium (DMEM)(Sigma-Aldrich) supplemented with 10% heat-inactivated fetal bovine serum (Atlanta Biologicals, Flowery Branch, GA, USA), 20 ng/mL epidermal growth factor (Invitrogen), 0.5 U/mL insulin (Eli Lilly, Indianapolis, IN, USA), 14 ng/mL glucagon (Bedford Laboratories, Bedford, OH, USA), 7.5 µg/mL hydrocortisone (Pharmacia Corporation, Kalamazoo, MI, USA) and 200 U/mL penicillin + 200 mg/mL streptomycin (Invitrogen). Following 30 min perfusion with medium, 50 to 100 million primary adult rat hepatocytes with over 90% viability were infused into the circuit in four subsequent injections at 10-min intervals as described previously [5]. The cells were recirculated in the system for 40 min at 25 °C. At the end of 40 min, the perfusate was collected, the viability of cells was determined by trypan blue exclusion.

### 2.7. Recellularized liver graft perfusion system

For perfusion culture experiments, the recellularized liver was transferred to a clean chamber; part of a perfusion system previously developed for whole-liver perfusion [20] that consisted of a peristaltic pump, bubble trap and an oxygenator. The system was placed in an incubator for temperature control (37 ºC), and the oxygenator was connected to a gas cylinder that contained atmospheric mixture. The medium was changed daily and cultures were maintained and assessed for 5 days.

### 2.8. Functional analysis

Perfusion culture medium samples were collected daily and analyzed for albumin content using enzyme linked immunosorbent assay as described previously [21] using a polyclonal antibody to rat albumin (Cappel Laboratories, Aurora, OH, USA). Urea content was measured with diacetylmonoxime with a commercially available kit (StanBio Laboratory, Boerne, TX, USA). The data reported were normalized to the initial cell seeding density.

### 2.9. Ex vivo diluted blood perfusion

Whole blood was freshly collected from female Lewis retired breeder rats and diluted in fresh PBS (1:1). The unmodified (DLM) or hDLM scaffold was connected to a custom-made perfusion system and perfused with 1:1 diluted blood ex vivo at a flow of 8 mL/min for 2 h. During perfusion, the portal pressure was measured using a water column attached to the inflow and used to estimate the resistance under constant flow. At the end of perfusion, the scaffolds were flushed with PBS, and examined macroscopically and imaged to determine the presence of blood clots that appeared as dark spots. The scaffolds were fixed and processed for histological analysis.

### 2.10. Preconditioning and heterotopic transplantation of (h)DLM

The median lobe of hDLM and DLM scaffolds was resected, recellularized, preconditioned and was subsequently transplanted heterotopically into syngeneic recipient rats. Unmodified DLM, 4BL and 8BL hDLM scaffolds were recellularized with 80 M primary rat hepatocytes and kept in perfusion culture for 24 h prior to transplantation. The engineered grafts were preconditioned with diluted whole blood prior to heterotopic transplantation. Whole blood was freshly collected from female retired breeder Lewis rats and diluted in fresh PBS (1:1) containing heparin (16.67 U/mL). The scaffold was connected to a custom–made perfusion system and perfused with 1:1 diluted blood ex vivo at 8 mL/min for 20 min. In the meantime, the recipient was prepared for transplantation. The abdomen was opened by midline incision and the intestine mobilized to the left, exposing the IHIVC. The right kidney was removed and the IHIVC was cross-clamped proximal and distal to the ligated renal vein over a ± 8–10 mm segment. The graft PV was then anastomosed to the recipient’s proximal IHIVC. Peripheral venous blood return ran from recipient proximal IHIVC and graft PV through the graft and out the graft’s hepatic veins into the graft SHIVC, which was anastomosed to the recipient’s distal IHIVC. Completion of the anastomoses was followed by reperfusion of the graft. After a gross examination of the graft for any evidence of clotting or bleeding, the recipient’s abdomen was closed. Twenty-four hours after surgery, the animals were anesthetized, and the grafts were harvested for histological analysis.

### 2.11. Histological analyses

hDLM and unmodified DLM scaffolds were fixed with 10% formalin, embedded in paraffin, and processed for staining with toluidine blue for heparin. Ex vivo blood perfused DLM scaffolds and recellularized liver grafts were fixed with 10% formalin, embedded in paraffin, and processed for staining with hematoxylin and eosin (H&E). Samples were imaged using Nikon Eclipse 800 and SPOT camera (Melville, NY, USA).

### 2.12. Statistical analysis

The statistical analysis of the data was performed using one- or two-way ANOVA and Student’s t-test with a confidence interval of 95%. Errors in figures and text represent standard error of the mean (SEM).

## 3. Results

### 3.1. Heparin Immobilization on DLM Scaffolds

**Figure 3.**
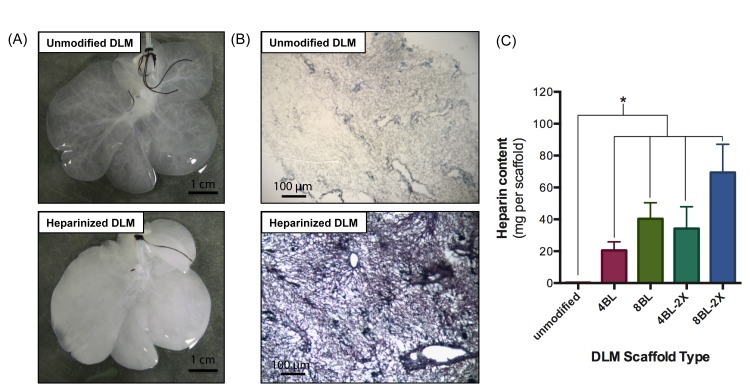
Characterization of heparinized DLM scaffolds. (A) Macroscopic images (B) Histological toluidine blue staining of unmodified and 4BL–2X heparinized DLM scaffolds. (C) Quantification of heparin content of the scaffolds after LbL deposition. Scale bars, (A) 1 cm, (B) 100 µm. * p = 0.0161

A series of heparin-immobilized scaffolds were prepared using layer-by-layer polyelectrolyte self-assembly method to improve DLM scaffold surface hemocompatibility (n=3/group). Heparin/PDADMAC bilayers were deposited via sequential perfusion of the oppositely charged polyelectrolyte solutions through the portal vein of the scaffold (Figure 2A–B). Macroscopically, the heparinized DLM scaffolds assumed a diffuse opaque appearance indicating the homogeneous presence of the polyelectrolyte multilayers throughout the scaffolds (Figure 3A). Qualitative evaluation of heparinized decellularized liver matrix (hDLM) scaffolds by toluidine blue staining confirmed successful deposition of heparin using the LbL technique (Figure 3B). Toluidine blue staining was positive in histological sections of hDLM scaffolds, while a minimal degree of staining was observed in unmodified DLM scaffolds. Quantitatively, the heparin content in the hDLM scaffolds varied with the number of bilayers and concentration of heparin used; ranging between 20.48 ± 5.45 (4BL) to 69.43 ± 17.66 mg (8BL–2X) (Figure 3C). The amount of heparin increased with increasing number of PDADMAC/heparin bilayers and with the increasing concentration of each polyelectrolyte solution. The heparin content in DLM without heparin (0 BL) was lower than all hDLM scaffolds, at 0.34 ± 0.03 mg per scaffold (p = 0.0161).

### 3.2. Recellularization of (h)DLM scaffolds

**Figure 4.**
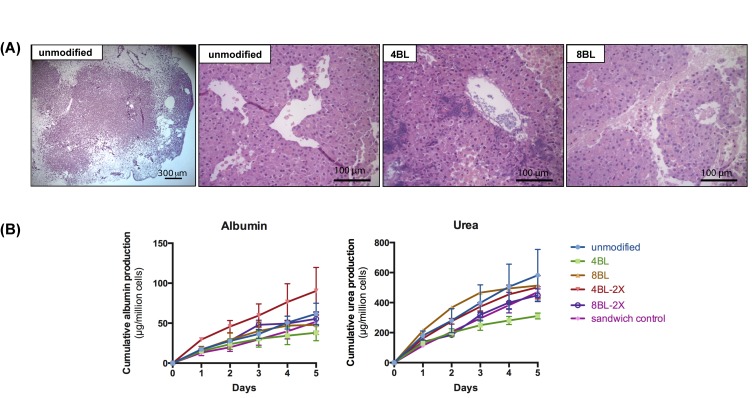
Recellularization of heparinized DLM scaffolds. A) Hematoxylin and eosin staining of unmodified and heparinized DLM scaffolds after 2 d in perfusion culture. (B) Cumulative albumin secretion and urea production by recellularized liver grafts over 5 d of perfusion culture. Scale bars (A) 300 µm; 100 µm.

The effect of heparin deposition on in vitro hepatocyte function was evaluated during perfusion culture of recellularized hDLM scaffolds (hDLM-R). Both DLM and hDLM scaffolds were repopulated with 80 million primary adult rat hepatocytes with an initial viability of 90% or higher. The recellularized scaffolds were maintained under perfusion through the portal vein for 5 days with daily medium changes. After 2 d of perfusion culture, histological analysis revealed that the grafts were repopulated with hepatocytes that formed plates around areas resembling vascular spaces, without obstructing the vessels. The cells assumed characteristic polygonal morphology and some of them were binucleated (Figure 4A). The morphological features of hepatocytes seeded in hDLM scaffolds were similar to those observed in unmodified DLM scaffolds.

All recellularized grafts displayed comparable levels of hepatic function as evidenced by the cumulative albumin and urea production over 5 d in perfusion culture (Figure 4B). Both the levels of albumin and urea production in heparinized grafts were similar to unmodified DLMs and controlled static plate cultures. These results suggest that the presence of LbL-deposited heparin on DLM scaffolds has no significant effect of hepatocyte viability and function.

### 3.3. Anticoagulation capacity of hDLM scaffolds

**Figure 5.**
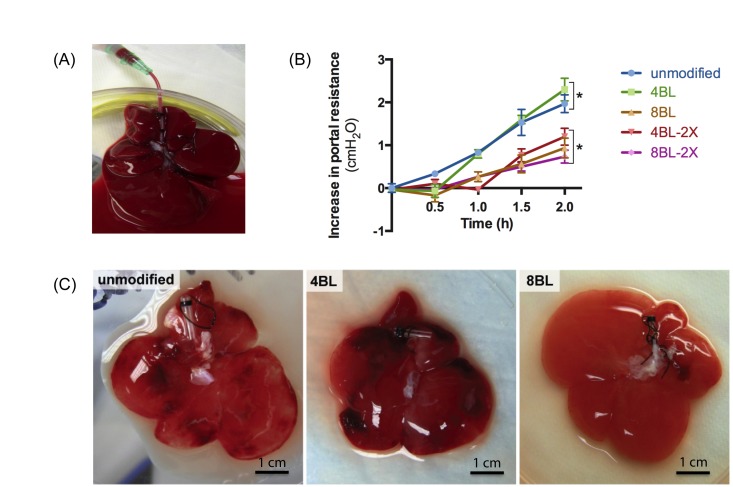
Ex vivo blood perfusion of heparinized DLM scaffolds. (A) Picture of ex vivo diluted whole-blood perfusion of (h)DLM. (B) The resistance of the scaffolds to blood flow at constant flow rates (8.0 mL/min). (C) The gross morphology of unmodified and heparinized scaffolds at the end of 2 h ex vivo blood perfusion. Scale bars (C) 1 cm. *p = 0.0013

As a first step towards assessing the potential of hDLM in preventing blood coagulation, we tested the hDLM scaffolds in ex vivo diluted whole blood reperfusion (Figure 5A). Flow rates were controlled and kept at 8 mL/min. Increased deposition of heparin in the 8BL, 4BL-2X and 8BL-2X hDLM scaffolds showed a lower increase in portal pressure than unmodified and 4BL scaffolds during the 2-h blood reperfusion with a statistically significant difference (p = 0.0013) (Figure 5B). At the end of 2 h of reperfusion the scaffolds were flushed with PBS leaving blood clots in unmodified and 4BL hDLM scaffolds macroscopically visible as dark spots throughout the lobe, while 8BL hDLM scaffolds exhibited minimal clotting (Figure 5C).

### 3.4. Transplantation of recellularized hDLM grafts

**Figure 6.**
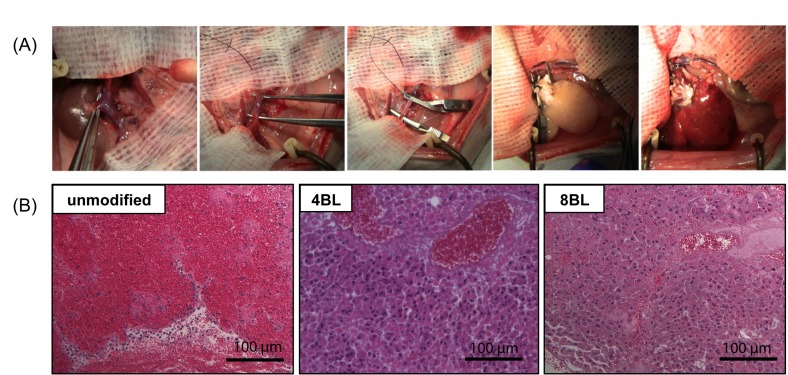
Heterotopic transplantation of heparinized DLM grafts. (A) Representative photographs during transplantation; left to right: First, right nephrectomy was performed (1), infrahepatic inferior vena cava was exposed (2), infrahepatic inferior vena cava was double clamped and dissected before anastomosis (3), the portal vein of the graft was anastomosed to distal stump of the recipient vena cava and the suprahepatic inferior vena cava of the graft was anastomosed to the recipient’s proximal stump of the vena cava (4). The engineered liver graft filled with blood after clamping off the recipient inferior vena cava (5). (B) H&E stain of transplanted liver grafts 24 h post-transplantation in unmodified (DLM-R), 4BL and 8BL grafts (hDLM-R). Scale bars: 100 µm.

The potential of recellularized hDLM grafts to extend the survival of recipient animals by delaying blood coagulation was assessed in a heterotopic liver transplantation model. To prime vascular patency pretransplantation, the grafts were preconditioned with diluted whole blood for 20 min. Unmodified, 4BL and 8BL DLM-R grafts (n=3/ group) were transplanted heterotopically in series with the IHIVC of the recipient following a right nephrectomy. The recellularized grafts reperfused with no significant bleeding (Figure 6A). Visually, both unmodified and hDLM-R led to mild congestion of the pre-graft IHIVC within 30 minutes of reperfusion. 24 h post-transplantation the recipient was reopened to assess the graft. At the time of harvest, all grafts were typically macroscopically patchy and small in size. Opening of the post-graft IHIVC revealed minimal outflow from the graft in both unmodified and heparinized samples. Unmodified grafts showed profuse intraparenchymal erythrocytes and thrombosis on histological analysis, while the 8BL DLM–R scaffold exhibited reduced thrombus formation in comparison to the recellularized unmodified DLM scaffold (Figure 6B).

## 4. Discussion

Tissue-engineered liver grafts offer the potential to overcome the dramatic shortage of donor livers for transplantation. Previous results have demonstrated that decellularized liver matrices, seeded with primary hepatocytes form a construct with sustained hepatocyte function in perfusion culture [5]. Overcoming the thrombogenicity of decellularized biological scaffold remains one of the major impediments to long-term in vivo survival following transplantation. While numerous studies have demonstrated that a preserved extracellular matrix provides an excellent scaffold for cell grafting, enabling cell-specific function in vitro perfusion culture, we have shown that the post-transplant survival remains limited to only 8 h [5]. Exposed matrix components induce an immunological and inflammatory response resulting in platelet activation and widespread thrombosis. Extensively treating the recipients with low molecular weight heparin and with Fab fragment of a chimeric monoclonal antibody, a glycoprotein IIb/IIIa inhibitor, to prevent ECM-related thrombosis led to hemorrhage [5]. Immobilized heparin has been explored in various applications to improve the compatibility of a coated surface with blood [7]. Bao et al. describes the immobilization of heparin to reduce coagulation in a tissue engineered liver, recellularized with hepatocyte spheroids, but not endothelial cells [15]. In a 90% hepatectomized rodent model, transplanted grafts were reported to support hepatic function for 72 hours with preservation of hepatocyte morphology and prolonged animal survival. Recently, the group reported the same technique, applied in a porcine model, where thrombosis was reduced during one hour of reperfusion after heterotopic implantation [16]. To improve graft hemocompatibility following blood reperfusion, we incorporated a similar heparin deposition strategy into our previously established decellularized liver matrix construct.

The results here show that layer–by–layer assembly of heparin-polyelectrolyte bilayers effectively deposits heparin in DLM, with a higher heparin content as the number of PDADMAC/heparin bilayers and polyelectrolyte concentration increases. Macro– and toluidine blue–stained microscopic analysis demonstrated effective uniform coverage throughout the liver. Heparin immobilization did not negatively impact seeding and function of primary hepatocytes during asanguinous in vitro culture of the recellularized hDLM. Ex vivo diluted whole-blood perfusion was used as a model for reperfusion, allowing controlled examination of thrombogenicity of the grafts. Post-perfusion flush of the (h)DLM allowed for macroscopic demonstration of thrombosis in the graft. 4BL hDLM grafts remained visually thrombogenic, with a similar distribution of clots as in the unmodified DLM, while an increased number of PDADMAC/heparin bilayers (8BL) resulted in a visually clot-free graft. Circulatory obstruction has been shown to lead to increases in vascular resistance during machine perfusion [22]. Higher efficiency of increasing number of BLs was confirmed by a dose–dependent increase in portal venous resistance; the lowest increase in resistance was observed in higher concentrations of heparin (4BL-2X, 8BL and 8BL–2X hDLM). To confirm this benefit in vivo, the hDLM-R was transplanted heterotopically and demonstrated reduced evidence of thrombosis and improved preservation of cellular distribution. However, flow remained obstructed in hDLM-R after 24 hours, with evidence of pre-graft congestion in all cases. Additionally, from the patchy and small appearance of the graft in all groups suggested we conclude that heparin immobilization did not improve transplantation suc-cess of DLM-R grafts.

An incomplete sinusoidal system results in a high resistance of the DLM graft, which is also seen in this work. The transplantation model employed here makes use of venous blood supply; with low–pressure caval venous perfusion forming a concern for satisfactory perfusion of the graft. An important difference between the in vivo and in vitro whole blood perfusion setting, which may underlie the discrepancy between the results, is the flow-driven perfusion in the latter. Achieving adequately low-resistance of the graft and in vivo reperfusion models that allow sufficient perfusion pressures require further optimization. Diluted whole-blood preconditioning by means of ex vivo machine perfusion, as presented here, may facilitate hepatocyte orientation around a patent, albeit immature, microvasculature, reducing resistance and pre-hepatic congestion and improving perfusion. Optimized seeding and endothelialization are also likely to promote microvascular development and have been shown to reduce resistance of the renal graft [23] and more recently of the porcine liver graft [24]. The latter study, which decellularized grafts to 20% residual DNA, reported that the structure of the sinusoidal system is sufficiently well retained for complete endothelialization. There is concern that more complete decellularization may compromise parenchymal structure and prevent complete endothelial coverage [25]. Further optimization of this approach is required. To determine initial feasibility, we previously showed that endothelial cells can be effectively seeded into the graft forming a functional sinusoidal system, which in conjunction with heparinization described here may improve the quality of the vascular system [5]. Additional approaches, such as elastin-like polypeptides to reduce thrombocyte adhesion [26] to further reduce thrombogenicity are also under investigation and may offer additional benefits.

In conclusion, LbL heparin deposition in decellularized liver matrices did not hinder the in vitro perfusion culture of DLM grafts and reduced resistance to ex vivo blood reperfusion with increasing levels of deposited heparin. However, successful transplantation of these constructs, as described in previous reports, was not achieved by this method. Further optimization including endothelialization of the matrix may offer the required hemocompatibility.
